# High-Throughput Screening and Identification of Human Adenovirus Type 5 Inhibitors

**DOI:** 10.3389/fcimb.2021.767578

**Published:** 2021-12-17

**Authors:** Xiaojing Wen, Li Zhang, Shan Zhao, Qiang Liu, Wenyi Guan, Jiajing Wu, Qiwei Zhang, Hongling Wen, Weijin Huang

**Affiliations:** ^1^ Division of Human Immunodeficiency Virus (HIV)/Acquired Immune Deficiency Syndrome (AIDS) and Sex-Transmitted Virus Vaccines, Institute for Biological Product Control, National Institutes for Food and Drug Control (NIFDC) and World Health Organization (WHO) Collaborating Center for Standardization and Evaluation of Biologicals, Beijing, China; ^2^ Department of Microbiological Laboratory Technology, School of Public Health, Cheeloo College of Medicine, Key Laboratory for the Prevention and Control of Infectious Diseases, Shandong University, Jinan, China; ^3^ Key Laboratory of China’s “13th Five-Year”, Shandong University, Jinan, China; ^4^ Guangdong Provincial Key Laboratory of Tropical Disease Research, School of Public Health, Southern Medical University, Guangzhou, China; ^5^ Guangdong Provincial Key Laboratory of Virology, Institute of Medical Microbiology, Jinan University, Guangzhou, China

**Keywords:** adenovirus, high-throughput screening, inhibitor, cardamomin, drug

## Abstract

Human adenovirus infections can develop into diffuse multi-organ diseases in young children and immunocompromised patients, and severe cases can lead to death. However, there are no approved antiviral drugs available to treat adenovirus diseases. In this study, a chemiluminescence-based, high-throughput screening (HTS) assay was developed and applied to screen human adenovirus 5(HAdV5)inhibitors from 1,813 approved drug library and 556 traditional Chinese medicine-sourced small-molecule compounds. We identified three compounds with *in vitro* anti-HAdV5 activities in the low-micromolar range (EC_50_ values 0.3-4.5 μM, selectivity index values 20-300) that also showed inhibitory effects on HAdV3. Cardamomin (CDM) had good anti-HAdV5 activity *in vitro*. Furthermore, three dilutions of CDM (150, 75, and 37.5 mg/kg/d) administered to BALB/c mouse models inhibited HAdV5-fluc infection at 1 day post-infection by 80% (p < 0.05), 76% (p < 0.05), and 58% (p < 0.05), respectively. HE-staining of pathological tissue sections of mice infected with a wildtype adenoviral strain showed that CDM had a protective effect on tissues, especially in the liver, and greatly inhibited virus-induced necrosis of liver tissue. Thus, CDM inhibits adenovirus replication *in vivo* and *in vitro*. This study established a high-throughput screening method for anti-HAdV5 drugs and demonstrated CDM to be a candidate for HAdV5 therapy, potentially providing a new treatment for patients infected with adenoviruses.

## Introduction

Adenoviruses, which were first discovered in 1953, are unencapsulated double-stranded DNA viruses that can cause zoonotic acute infectious diseases. They usually invade the respiratory system, digestive system, urinary system, or nervous system and cause a number of diseases (Dhingra et al., 2019). Infections show various tissue tropisms depending on the type of adenovirus: types 3, 4, 5, and 7 mainly infect the respiratory tract and eyes, while types 40, 41, and 52 affect the gastrointestinal tract (Lion, 2014; Prusinkiewicz and Mymryk, 2019). Adenovirus infections are globally distributed and may occur in any season. Since their discovery, adenovirus outbreaks have been reported both in China and abroad. China has not yet established a nationwide epidemiological surveillance program for adenovirus infections, and the CDC is unable to detect or provide early outbreak warnings (Bailey et al., 2018).

Currently, there are no approved medications for adenoviral infections, and the broad-spectrum antiviral drugs used clinically are not very effective against these pathogens (Martinez-Aguado et al., 2015). Studies have shown that cidofovir has antiviral activity against all adenovirus species but has low oral utilization, obvious renal toxicity, and cannot offer long-term protection (Wold and Toth, 2015). A lipid-related derivative of cidofovir, brincidofovir, is currently in the clinical trial stage, has good application prospects for the treatment of adenoviral diseases, and is not nephrotoxic (Ramsay et al., 2017). Vaccines against type 4 and 7 adenoviruses are currently only used in the U.S. military and are not available to the general population (Binder et al., 2017). Therefore, there is an urgent need to develop new, highly efficient vaccines and drugs against adenovirus infections.

The development of new antiviral drugs is slow, expensive, and the success rate is low. Compared with the development of new drugs, investigating the potential anti-viral functions of previously approved drugs, i.e., drug repurposing, may be more effective and efficient. Approved drugs have the advantages of known pharmacology and toxicity profiles, established safety in humans, and proven manufacturing and formulation feasibility. Drug repurposing has additional advantages, such as a low risk of failure, short development time, and low cost of consumption (Sohraby et al., 2017; Mercorelli et al., 2018; Pushpakom et al., 2019).

In this study, we developed a chemiluminescence-based high-throughput screening (HTS) assay for the discovery of human adenovirus 5(HAdV5) antiviral inhibitors and screened a library of 1,813 FDA-approved drugs and 556 traditional Chinese medicine-sourced small molecule compounds. We aimed to identify compounds with antiviral effects against HAdV5.

## Materials and Methods

### Cell Lines and Virus

We cultured 293T cells (ATCC, CRL-3216) and A549 cells (ATCC, CCL-185) in high-glucose Dulbecco’s Modified Eagle’s Medium (HyClone, South Logan, UT) supplemented with 10% FBS (Gibco, Carlsbad, CA), 1% penicillin–streptomycin solution (Gibco), and 2% 4-(2-hydroxyethyl)-1-piperazineethanesulfonic acid (Gibco) in 5% CO_2_ at 37°C. The cells were passaged every 2-3 days.

The HAdV5–fluc virus was purchased from Shandong Weizhen Company. HAdV3-GFP, HAdV4-GFP, andnHAdV7-GFP were stored in a P3 Laboratory of the School of Public Health, Southern Medical University, Guangzhou, China. HAdV14-GFP was a gift from the Institute of Respiratory Diseases, Guangzhou Medical University. HAdV55-GFP was a gift from the Guangzhou Institutes of Biomedicine and Health, Chinese Academy of Sciences.

### Compound Library

The 1,813 approved drug compounds were purchased from TargetMol (Approved Drug Library L1000), and the 556 traditional Chinese medicine-sourced small molecule compounds were provided by the National Standard Chemical Control Library of NIFDC(110701-112025). The compounds were dissolved to 10 mM in dimethyl sulfoxide (Sigma-Aldrich, St. Louis, MO) and stored at −80°C until further use.

### Screening Assay With HAdV5–fluc

To test whether our system met the HTS conditions, Wells in columns 1 and 2 of the 96-well assay plates contained 293T cells as a control (0% response), whereas HAdV5 was added to all other wells (positive control, 100% response). The results were observed 24 h later. The signal-to-basal (S/B) ratio (S/B=mean signal/mean background), Z factor(Z=[1-3SD of sample +3SD of control]/|mean of sample - mean of control|) and CV were caculated. In the first round of HTS, 293T cells were seeded into 96-well plates at a density of 3 × 10^4^ cells/well (Corning, Inc., NY, USA). After overnight incubation, the cells were treated with the 10 μM compounds in duplicate, then incubated at 37°C, 5% CO_2_ for 1 h. HAdV5–fluc (2.4×10^4^ plaque-forming units [PFU]/mL, MOI=0.67) was added to each well(50 μl), and luciferase activity was measured 23 h later. In short, 100 μl of culture medium was gently aspirated, and 100 μl of Bright-Glo luciferase reagent (Promega, Madison, WI) was added to each well to react with the cells for 2 min at room temperature. Luminescence was then measured using a GLOMAX 96 microplate luminometer (Promega). The percentage inhibition was calculated as 100 × [1 − (RLU in the presence of compound − RLU of negative control)/(RLU of positive control − RLU of negative control)](Negative control refers to the group of cells without drugs and viruses). The primary hits showed no apparent cytotoxicity and a >50% decrease in adenovirus replication in duplicate wells with 10 µM compounds. The 50% effective concentration (EC_50_) was determined from the dose–response curve of HAdV5–fluc at eight serially diluted doses, 90–0.04 µM, in the second screening.

### Verification assay with HAdV3-GFP, HAdV4-GFP, HAdV7-GFP, HAdV14-GFP, and HAdV55-GFP

A549 cells were seeded at a density of 2 × 10^4^ cells/well into 96-well plates. The following day, the medium was replaced 2 h in advance of adding the indicated concentration of drug. The drug concentrations used were as described above. Four replicate wells were used for each drug concentration, and 50 μl of virus (4 × 10^5^ PFU/mL) was added to each well. The plates were incubated in a shaker for 30 min, and the medium was changed to the maintenance medium with the corresponding concentration of drug after 2 h. After 48 h, three fluorescence images (Nikon eclipse TE2000-U)were randomly recorded per well. After photographing, the GFP fluorescence intensity of HAdV-GFP in cells was analyzed by imagJ software. Five photos were taken here and three were analyzed after removing the highest value and the lowest value.

### Cytotoxicity Assay

Cytotoxicity testing was performed using the CellTiter Glo luminescent cell viability assay kit (Promega) to determine the 50% cytotoxic concentration (CC_50_) of each test compound in the absence of the virus. Serial drug dilutions, from 90 to 0.04 μM, were added to the 293T cells in 96-well plates, then 50 μL of complete medium was added instead of the virus. After incubation at 37°C and 5% CO_2_ for 24 h, cell viability was measured using the GLOMAX 96 microplate luminometer.

### Confirmation of HAdV5 Inhibitors Using an *In Vivo* Bioluminescence Imaging Model

Mice were handled in accordance with institutional (SCXK(jing) 2017-0005)NIFDC, Beijing, China) guidelines for laboratory animal care and use, and the Animal Care and Use Committee at the NIFDC approved the study protocol. Four to five-week-old female BALB/c mice (n = 5 per group) were obtained from the Institute of Laboratory Animal Resources of NIFDC. To challenge the mice, 4.5 × 10^6^ PFU HAdV5 were administered *via* the tail vein to groups of five mice. Cardamomin (CDM) was dissolved in 0.5% methylcellulose (Sigma-Aldrich, St. Louis, MO) and aliquoted into three doses (150 mg/kg, 75 mg/kg, and 37.5 mg/kg), which were IP-injected into three groups of mice. The drug was administered 6 h before infection, then once daily until 5 dpi. Bioluminescence imaging (BLI) analysis was performed with the IVIS Lumina Series III Imaging System (PerkinElmer, Baltimore, MD) as previously described (Zhang et al., 2017). We used Living Image software (Caliper Life Sciences, Baltimore, MD) to analyze the regions of interest, which were represented by the total flux in photons per second.

### *In Vivo* Inhibition

Four to six-week-old male BALB/c mice (n 10 per group) were divided into three groups: A, drug control group; B, drug and virus experimental group; and C, virus control group. We inoculated 6.4 × 108 TCID50 of HAdV-5 to mice *via* the tail vein and CDM (125 mg/kg) was administered by IP injection 6 h before infection, then once a day until 5 dpi. The mice were weighed daily and the weights recorded and were euthanized at 1, 3, 5, 8 dpi.The livers, spleens, and lungs of mice that died of natural causes or were sacrificed daily were divided into three parts, which were used for pathological sectioning, immunohistochemical observation, and molecular analysis. For the molecular analysis, virus DNA was extracted by QIAamp DNA Mini Kit(Qiagen, Germany), and detected by Quantitative Real-time PCR (Zhang et al., 2020). For the pathological sectioning and immunohistochemical observation, tissue sections were embed in paraffin after washed with PBS three times. pathological sectioning by Hematoxylin-eosin (HE) staining and immunohistochemical by streptavidin-biotin peroxidase technique were performed respectively. A rabbit neutralizing antibody against HAdV5 was used for immunohistochemistry and the dilution ratio is 1:50.

### Statistical Analysis

GraphPad Prism 6 (San Diego, CA, USA) was used to analyze the EC_50_ and CC_50_ values of the compounds and the bioluminescence imaging data. The statistical comparisons were performed using the t-test and ANOVA in IBM SPSS Statistics 24.0. The values α = 0.05 and P < 0.05 were considered statistically significant.

## Results

### Identification of HAdV5 Inhibitors Using an *In Vitro* Screen

**Figure 1:**
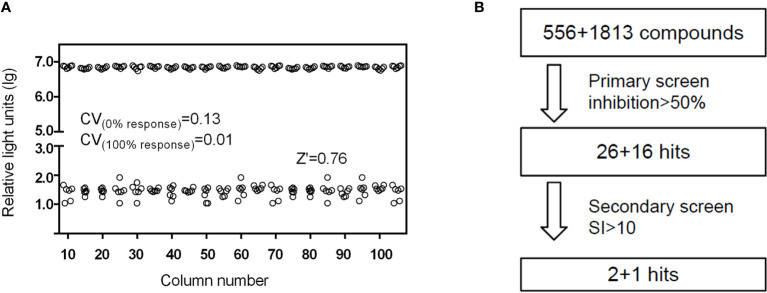
**(A)** Scatter plot of DMSO plate screening results. Wells in columns 1 and 2 of the 96-well assay plates contained 293T cells as a control (0% response), whereas HAdV5 was added to all other wells (positive control, 100% response). The results were observed 24 h later. **(B)** Flow chart of HTS assay using HAdV5. In the primary screen, 293T cells were treated with 10 μM compounds in duplicate, and the virus was added after mixing the cells and compounds for 1 h. The luciferase activity was measured 23 h later. In the second screen, the drugs were diluted into eight serially diluted doses, 90–0.04 µM. Others operation were the same as before.

**Figure 2:**
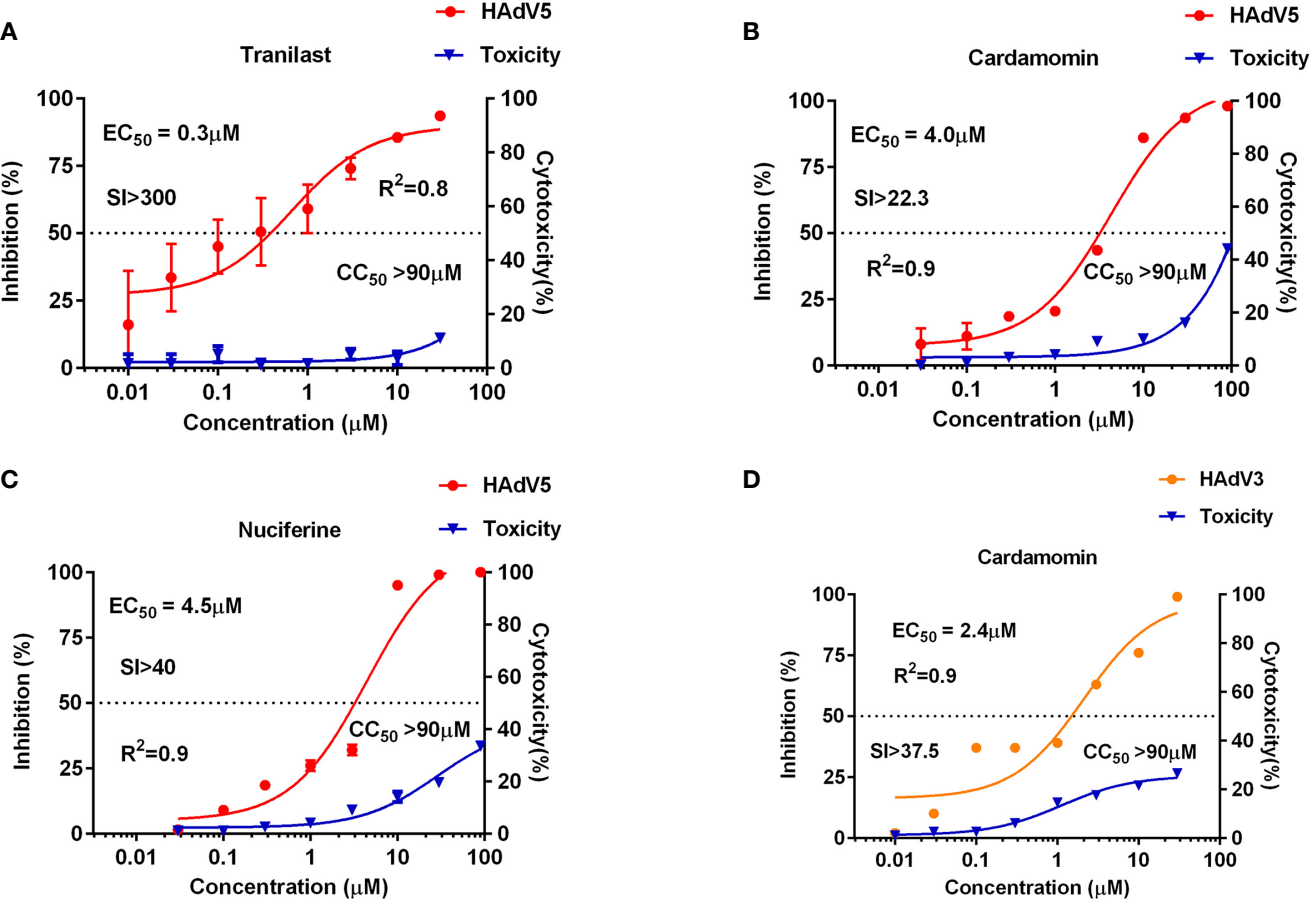
Anti-HAdV3/5 activities of three candidate compounds. The 293T cells (3 × 10^4^ cells/well) were infected with HAdV5 and treated for 24 h with eight doses of tranilast **(A)**, cardamomin **(B)**, and nuciferine **(C)**. A549 cells (3×10^4^ cells/well) were infected with HAdV3 and treated for 24 h with eight doses of cardamomin **(D)**. Antiviral activity of HAdV3/5 is shown in red, and cytotoxicity is shown in blue.

Using HAdV5-fluc, we applied HTS to identify inhibitors of viral entry from 1,813 approved drug library and 556 traditional Chinese medicine-sourced small molecule compounds. The HTS conditions of cell-seeding density and HAdV5 dose were optimized at 30,000 cells/well and 1 × 10^3^ PFU/well, respectively. Under these conditions, the signal-to-basal (S/B) ratio and Z-factor were 209,321 and 0.76, respectively (**Figure 1A**). The above data indicates that the experiment met HTS conditions (Zhang et al., 1999; Zhang et al., 2006). The HTS schematic is depicted in **Figure 1B**. The primary screen was performed for a single dosage (10 µM) of each compound to identify those with activity against HAdV5. The results showed 42 compounds resulted in >50% inhibition, thus necessitating a second confirmation round. Each of the 42 compounds was diluted at a 1:3 ratio to generate four concentrations ranging from 90 µM to 0.04 µM to confirm their activity against HAdV5 and determine their EC_50_ values. This evaluation of antiviral activity together with cytotoxicity was critical because compounds with cytotoxicity can appear to elicit antiviral activity, while the cellular inhibition assay is largely dependent on cell viability. Drugs with a dose-response relationship were further evaluated in parallel with cytotoxicity in uninfected 293T cells using eight-point dose response curves (starting from 90 µM) to generate EC_50HAdV5_ and CC_50_ values. The ratio of CC_50_ and EC_50HAdV5_ was calculated for use as a selectivity index (SI). After the final reconfirmation screening, three compounds: Tranilast, Cardamomin and Nuciferine were confirmed as possessing efficacious inhibition effects against HAdV5, low cytotoxicity, and SI values above 10 (**Table 1** and **Figures 2A–C**).

**Table 1:** *In vitro* anti-HAdV5 activity of three candidate compounds.

Compound Name	EC_50_-HAdV5 (µM)	CC_50_ (µM)	SI=CC_50_/EC_50HAdV5_
Tranilast	0.3	>90	>300
Cardamomin	4.0	>90	>22.5
Nuciferine	4.5	>90	>20

The EC_50_ was determined by inhibition assays in 293T cells infected with HAdV5. Each value is the average of three experiments. The CC_50_ was determined using the CellTiter-Glo Luminescent Cell Viability Assay. Selectivity Index: ratio of CC_50_/EC_50_.

The three drugs screened for HAdV5 inhibition using 293T cells were further tested for their effects on several other types of adenovirus: HAdV3, HAdV4, HAdV7, HAdV14, and HAdV55. Wildtype adenoviruses fused with GFP (AdV3-GFP, HAdV4-GFP, HAdV7-GFP, HAdV14-GFP, and HAdV55-GFP), which corresponding to HAd5-fluc were used for detection. The results show that CDM had an inhibitory effect on HAdV3-GFP but not on other types of adenoviruses at the cellular level. The result of CDM was showed in **Figure 2D**.

### CDM Acted as HAdV5 Inhibitor in the BLI Model

**Figure 3:**
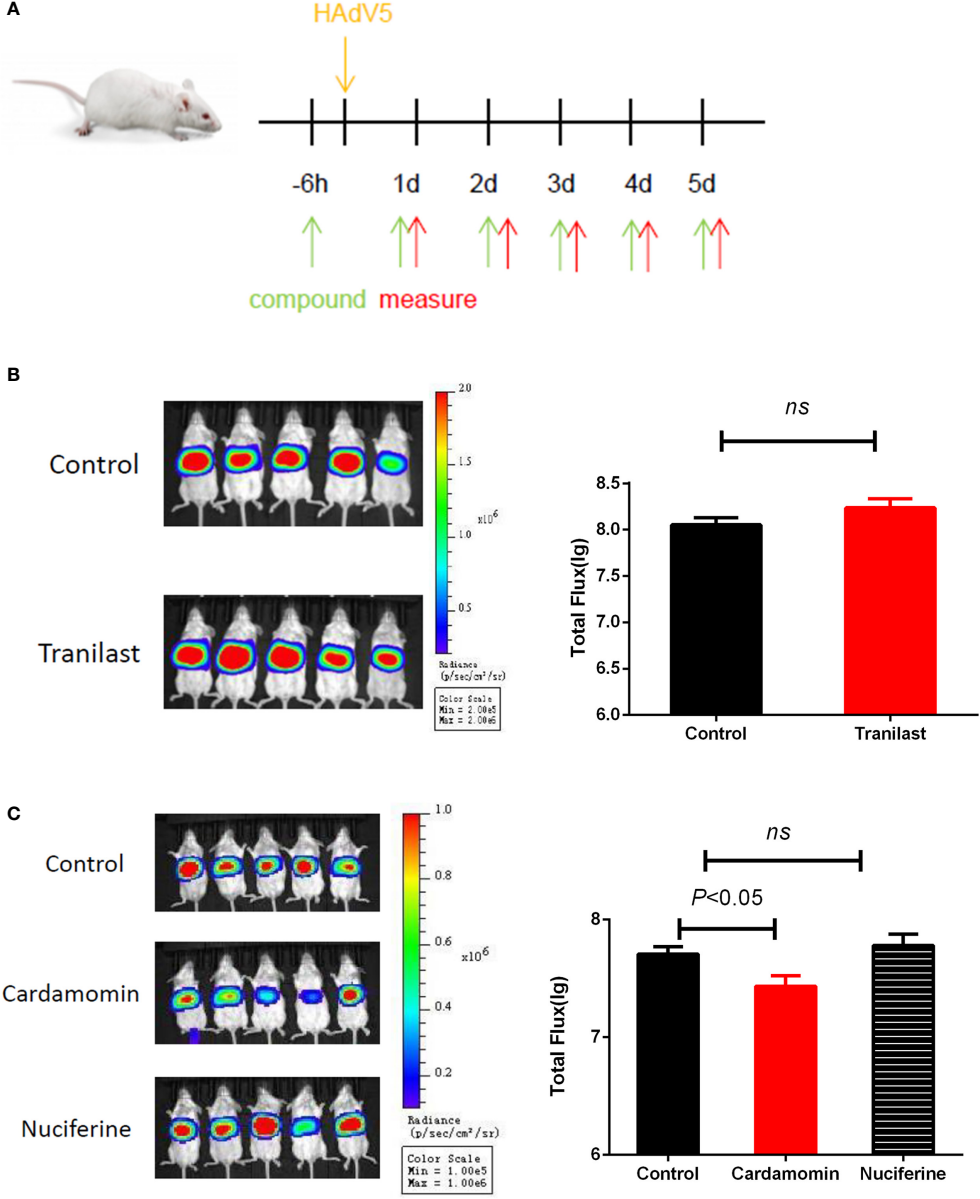
**(A)** Schematic representation *in vivo* screening.Primary *in vivo* screen of tranilast. Four to five-week-old female BALB/c mice were infected *via* the tail vein with 4.5 × 10^6^ PFU HAdV5 (0 h). **(B)**Tranilast (75 mg/kg/d), **(C)** Cardamomin and nuciferine (75 mg/kg/d)was delivered by IP injection to Balb/c mice 6 h before infection and re-administered at 1,2,3,4,and 5 after infection. Bioluminescence data were collected 1 day after infection. Total flux (photons/s) from luciferase expression in HAdV5 was measured using a bioluminescence imaging method. The statistical comparisons were performed using the t-test. ns, not significant.

**Figure 4:**
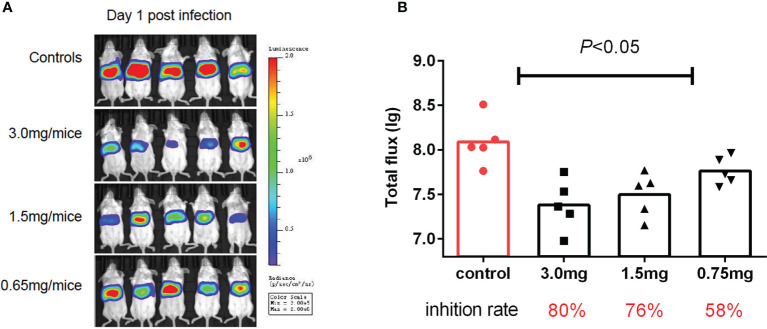
Second cardamomin screen. **(A)** Cardamomin (150 mg/kg/d, 75 mg/kg/d, and 32.5 mg/ kg/d) was delivered by IP injection to Balb/c mice 6 h before infection and re-administered at 1,2,3,4, and 5 after infection. Bioluminescence data were collected 1 day after infection. **(B)** Total flflux (photons/s) from luciferase expression in HAdV5 was measured using a bioluminescence imaging method. The statistical comparisons were performed using the ANOVA.

To test if the antiviral activities of the active compounds observed *in vitro* were translatable to *in vivo* HAdV5 infection, we evaluated the treatment effects of the three candidate compounds from the HTS. First, the three compounds (75 mg/kg/d) were delivered by IP injection into BALB/c mice 6 h before infection. The mice were infected with 4.5 × 10^6^ PFU HAdV5 (0 h) *via* the tail vein, and the drug was re-administered at 1,2,3,4 and 5 dpi. Based on the BLI analysis, we concluded that CDM treatment of mice resulted in decreased HAdV5 infection. The inhibitory effect was demonstrated by the reduced BLI signal compared with the control group. The other compounds did not show infection inhibition effects *in vivo* (**Figure 3**). The experiment was subsequently repeated; CDM was administered in three doses, 150 mg/kg/d, 75 mg/kg/d, and 32.5 mg/kg/d, which exhibited obvious effects, with inhibitory rates of 80% (p < 0.05), 76% (p < 0.05), and 58% (p < 0.05), respectively (**Figure 4**).

### Inhibition *In Vivo* Verified With Wild Type HAdV5

From the above results, CDM had a clear inhibitory effect on HAdV5-infected mice. A wildtype HAdV5 strain was used for further verification. The efficacy of the drug was evaluated by observing changes in virus DNA content in tissues, HE-staining, and immunohistochemical staining. There is no obvious change in DNA content in tissues.

**Figure 5:**
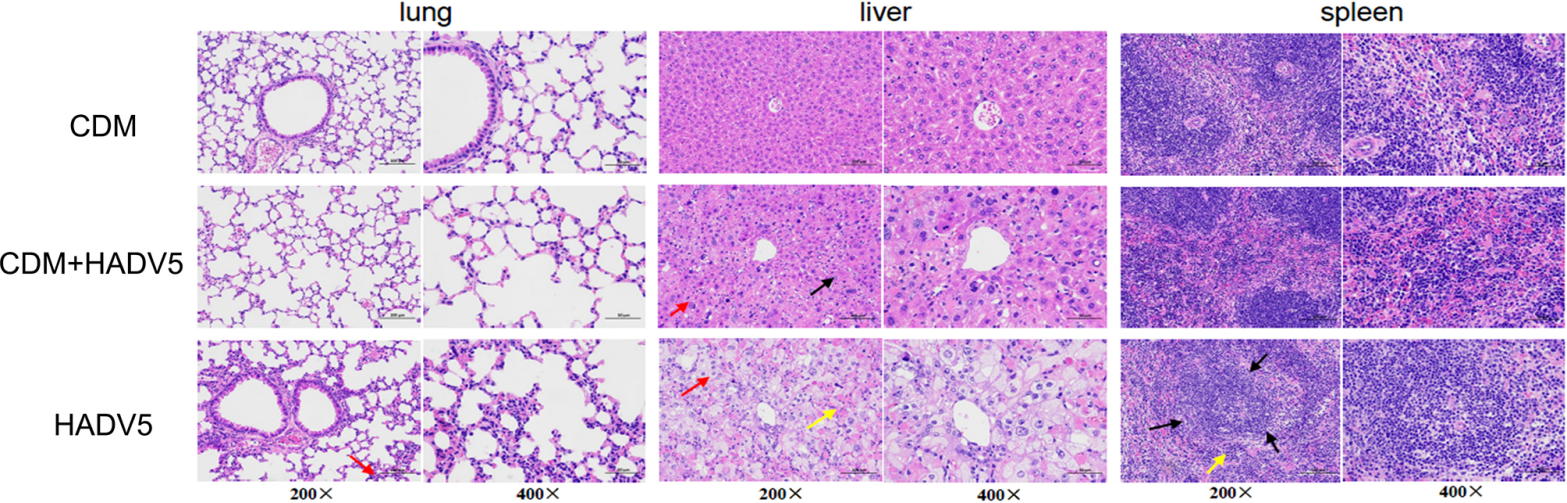
Hematoxylin-eosin (HE) staining of lung, liver, and spleen tissue of mice at 5 dpi. Balb/c mice were intraperitoneally (IP) injected with 150 mg/kg/d cardamomin for 6 days (D0-D5) continuously and challenged 6 h after the first IP injection. The experimentl group (CDM+HAdV5) was injected with HAdV5 3.2 × 10^10^ TCID_50_/kg, and the drug control group (CDM) was injected with an equal volume of PBS. Tissues were collected on day 5. Alveolar wall granulocyte infiltration (red arrow) was more obvious and severe in the virus control group (HAdV5). The red and white pith were less clearly demarcated in the spleen tissue of the virus control group (black arrow), and a small amount of red pulp caused congestion in the medulla sinuses, and a small number of extramedullary hematopoietic cells were seen (yellow arrow).

Infected lung, liver, and spleen tissues were collected at 1-5 dpi for HE staining analysis. We compared the results for the control group and the experimental group at 1-4 dpi, and no significant differences were observed. On the fifth day, clear organizational differences were noted. The results are shown in **Figure 5**.

At 5 dpi, the bronchial and alveolar walls of the lung tissue were not clearly abnormally structured in the three groups. Local alveolar wall thickening was observed in both the experimental and virus control groups. Alveolar wall granulocyte infiltration was more obvious and severe in the virus control group. The lung tissue damage in the virus control group(HAdV5) was more serious than that in the experimental group(CDM+HAdV5).

The liver lobules of mice in the drug control group were intact, the liver cells were arranged in a regular order, the liver cord was structured normally, and we observed no obvious inflammatory reaction. In the experimental group, spot-like necrosis of hepatocytes, balloon-like changes in some hepatocytes, swelling of the cell body, and loose staining of the cytoplasm (red arrow) were observed. Compared with the other two groups, the virus control mice showed more severe liver necrosis. Balloon-like hepatocytes were widespread, and there was abundant hepatocellular necrosis, cytoplasmic eosinophilic enhancement (yellow arrow), and a small amount of inflammatory cells infiltrate. Therefore, the liver tissue of the experimental group was less damaged than that of the virus control group.

In the control group, the red and white pith were clearly demarcated, and red pith lymphocytes were abundant. In the experimental group, the red and white pith were clearly demarcated, the germinal center was not obvious, and red pith lymphocytes were abundant. The red and white pith were less clearly demarcated in the spleen tissue of the virus control group. A small amount of red pulp caused congestion in the medulla sinuses, and a small number of extramedullary hematopoietic cells were seen (yellow arrow). Therefore, the degree of spleen tissue damage was slightly less in the experimental group than the virus control group. In summary, CDM had a protective effect on the tissues of mice infected with adenovirus.

**Figure 6:**
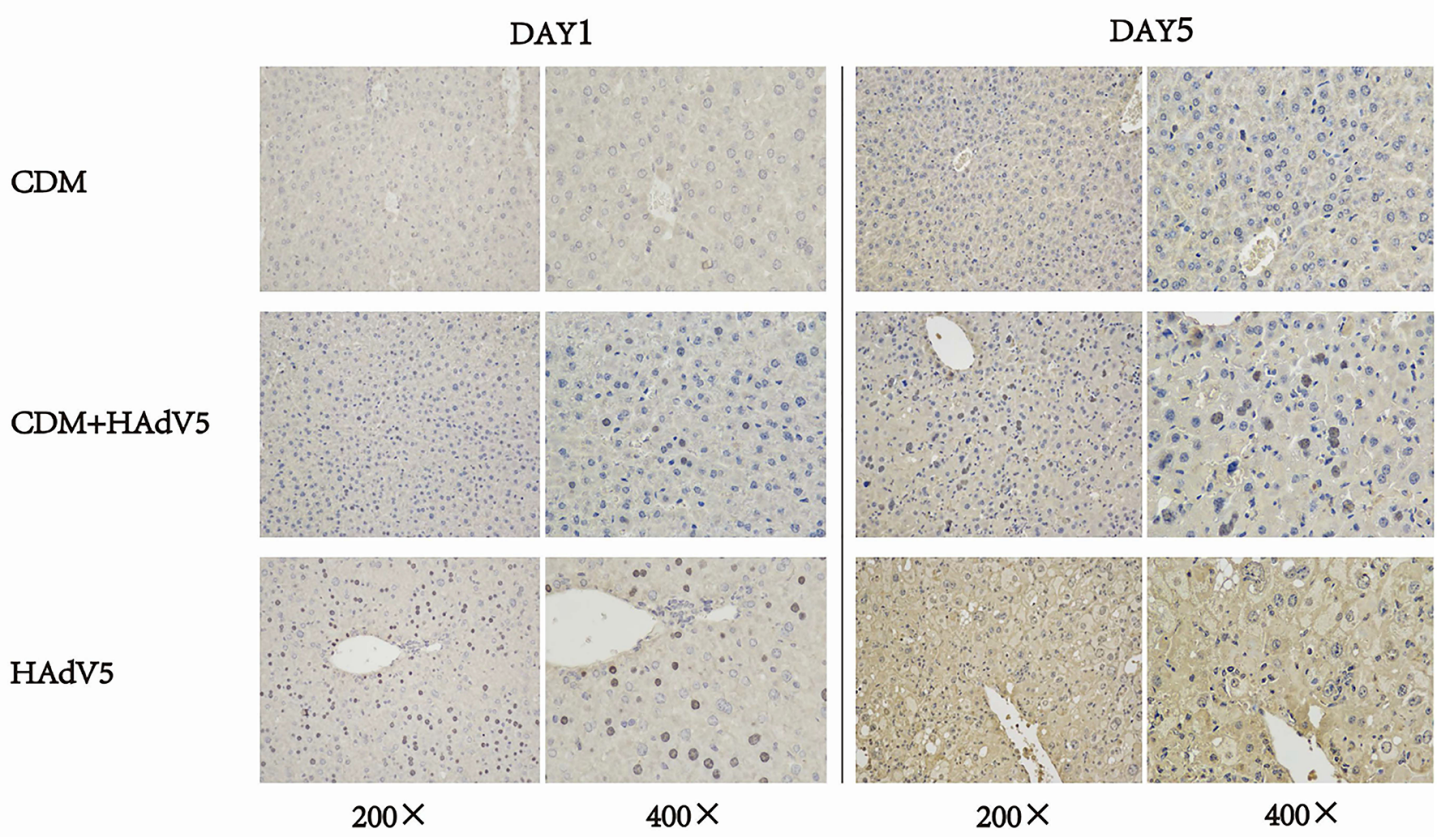
Immunohistochemical staining of mouse liver tissue at 1 and 5 dpi. The experiment group (CDM+HAdV5) has less positive staining than virus control group (HAdV5) that means the experiment group has fewer adenovirus capsid proteins. BALB/c mice were intraperitoneally injected with 150 mg/kg/d cardamomin for 6 days (D0-D5) continuously and challenged 6 h after first IP injection. The experimental group was injected with HAdV5 3.2 × 10^10^ TCID_50_/kg, and the control group (CDM) was injected with an equal volume of PBS. Tissues were collected on days 1 and 5.

We also performed immunohistochemical analyses using a rabbit neutralizing antibody dilution of 1:50 against HAdv5 a group of proteins expressed by adenoviruses—adenovirus capsid proteins. Immunochemical staining images of the liver at 1 and 5 dpi are shown in **Figures 6**. The liver tissue of the experimental group was weakly positive for the virus on day 1 and was slightly more positive than the drug control group; however, the control group showed more positive staining at 5 dpi. According to the liver tissue immunohistochemical results on 5 dpi, CDM had a certain inhibitory effect on the virus.

## Discussion

The aim of this study was to identify compounds that can act as effective agents against HAdV5 infection by establishing a high-throughput antiviral drug screening method. We screened 1,813 approved drugs and 556 traditional Chinese medicine-sourced small molecule compounds and identified one candidate compound, CDM. We demonstrated that CDM has activity against HAdV5 and HAdV3 *in vitro* and can inhibit HAdV5 infection in BALB/c mice. CDM, which is a traditional Chinese medicine small molecule compound from the ginger plant, has been gaining interest from the scientific community because of its effect on human health, and there has been an increasing number of studies conducted on its properties, such as anti-inflammatory, anti-tumor, antioxidant, and other activities (Zhang et al., 1999; Zhang et al., 2006; Jin et al., 2019). CDM has been demonstrated to exert an anti-inflammatory effect by inhibiting the expression of toll-like receptor 4 and inactivating NF-κB and the mitogen- activated protein kinase pathway (Ren et al., 2015).

In the current experiments, CDM was administered to model mice 6 h before infection and once daily until 5 dpi, and the compound inhibited infection by up to 80%. Therefore, CDM may be useful as a preventive medication for HAdV5 infection. The other identified compounds did not show inhibition of viral infection *in vivo*. Interestingly, tranilast has been previously reported to have anti-smallpox activity (Wu et al., 2019), and it presented the highest anti-HAdV5 potency *in vitro* (SI > 75), but the compound did not show the expected efficacy *in vivo*. Several factors, such as the type of formulation, drug-enzyme interactions, or even interspecies differences in gastrointestinal adsorption, may affect drug efficacy. According to Geisbert, many drugs work in cells but have no effect in rodents, or have effect in rodents but not in monkeys (Enserink, 2014). Interestingly, CDM only inhibited HAdV5 and HAdV3 genotypes, but not HAdV4, HAdV7, HAdV14, and HAdV55. This suggests that the antiviral mechanism of CDM is related to differences between adenovirus types. The protein difference in different adenovirus types may affect the antiviral effect of CDM. The reasons for differences in drug effects among different species and the underlying mechanisms need to be further explored.

To further understand the anti-HAdV5 mechanisms of CDM, we performed *in vivo* validation with a wildtype strain of HAdV5. HAdV-5 can infect mice, and the virus protein can be expressed in mice. However, no infectious progeny viruses could be produced. Therefore, we cannot examine whether CDM can influence the replication of HAdV5. As seen from HE-staining of murine pathological tissue (**Figure 5**) at 5 dpi, CDM had a significant protective effect against inflammation and necrosis, especially in the liver, as it greatly inhibited liver tissue necrosis caused by the virus. Therefore, the drug may play a more significant role in virus protein expression and tissue protection.

Furthermore, CDM has been reported to have antiviral efficacy against HIV-1 and dengue virus type 2. CDM was effective against HIV-1 with EC_50_ at 115 μM, and it acts on a key enzyme in the life cycle of HIV-1: HIV-1 protease. CDM inhibits the dengue virus by up to 71% at 1.5 mM. The antiviral activity of CDM may be related to its activity on virus-associated proteases (Kiat et al., 2006; Goncalves et al., 2014). Another study showed that CDM can prevent the G2/M phase of the cell cycle (Nawaz et al., 2020).Whether the antiviral mechanisms of CDM are related to this effect requires confirmation by relevant experiments. This study provided the basis for studying anti-adenovirus drugs. However, further research is needed on the mechanisms of CDM’s effect on adenoviruses.

## Conclusions

To identify novel anti-Ad5 inhibitors, we established a high-throughput cell screening method and screened 1,813 approved drugs and 556 traditional Chinese medicine-sourced small molecule compounds. Three candidate compounds were found to be effective anti-Ad5 agents at the cellular level, but only CDM was effective both *in vitro* and *in vivo*. The efficacy of CDM against Ad5 has not been reported previously. It is a potential new anti-Ad5 drug, and we expect these efforts will lead to the development of new antiviral drugs that are efficacious against adenovirus.

## Data Availability Statement

The raw data supporting the conclusions of this article will be made available by the authors, without undue reservation.

## Ethics Statement

The animal study was reviewed and approved by the Animal Care and Use Committee at the National Institutes for food and drug control. Written informed consent was obtained from the owners for the participation of their animals in this study.

## Author Contributions

QZ and HW conceived, designed, and supervised the experiments. XW, HW and LZ wrote the manuscript. XW, QL, WG, SZ, and JW performed the experiments. All authors contributed to the article and approved the submitted version.

## Funding

This work was supported by National Science and Technology Major Projects of Infectious Disease [grant number 2018ZX10731101].

## Conflict of Interest

The authors declare that the research was conducted in the absence of any commercial or financial relationships that could be construed as a potential conflict of interest.

## Publisher’s Note

All claims expressed in this article are solely those of the authors and do not necessarily represent those of their affiliated organizations, or those of the publisher, the editors and the reviewers. Any product that may be evaluated in this article, or claim that may be made by its manufacturer, is not guaranteed or endorsed by the publisher.
